# Genomic Aberrations in Crizotinib Resistant Lung Adenocarcinoma Samples Identified by Transcriptome Sequencing

**DOI:** 10.1371/journal.pone.0153065

**Published:** 2016-04-05

**Authors:** Ali Saber, Anthonie J. van der Wekken, Klaas Kok, M. Martijn Terpstra, Lisette J. Bosman, Mirjam F. Mastik, Wim Timens, Ed Schuuring, T. Jeroen N. Hiltermann, Harry J. M. Groen, Anke van den Berg

**Affiliations:** 1 Department of Pathology and Medical Biology, University of Groningen, University Medical Center Groningen, Groningen, Netherlands; 2 Department of Pulmonary Diseases, University of Groningen, University Medical Center Groningen, Groningen, Netherlands; 3 Department of Genetics, University of Groningen, University Medical Center Groningen, Groningen, Netherlands; University of North Carolina School of Medicine, UNITED STATES

## Abstract

*ALK*-break positive non-small cell lung cancer (NSCLC) patients initially respond to crizotinib, but resistance occurs inevitably. In this study we aimed to identify fusion genes in crizotinib resistant tumor samples. Re-biopsies of three patients were subjected to paired-end RNA sequencing to identify fusion genes using deFuse and EricScript. The IGV browser was used to determine presence of known resistance-associated mutations. Sanger sequencing was used to validate fusion genes and digital droplet PCR to validate mutations. *ALK* fusion genes were detected in all three patients with *EML4* being the fusion partner. One patient had no additional fusion genes. Another patient had one additional fusion gene, but without a predicted open reading frame (ORF). The third patient had three additional fusion genes, of which two were derived from the same chromosomal region as the *EML4*-*ALK*. A predicted ORF was identified only in the *CLIP4*-*VSNL1* fusion product. The fusion genes validated in the post-treatment sample were also present in the biopsy before crizotinib. *ALK* mutations (p.C1156Y and p.G1269A) detected in the re-biopsies of two patients, were not detected in pre-treatment biopsies. In conclusion, fusion genes identified in our study are unlikely to be involved in crizotinib resistance based on presence in pre-treatment biopsies. The detection of *ALK* mutations in post-treatment tumor samples of two patients underlines their role in crizotinib resistance.

## Introduction

Chromosomal rearrangements involving the anaplastic lymphoma kinase (*ALK*) gene can occur in different cancers including NSCLC, anaplastic large cell lymphoma and inflammatory myofibroblastic tumors [1]. The echinoderm microtubule-associated protein-like 4 (*EML4*) gene is the most common fusion partner of the *ALK* gene in NSCLC [2]. Presence of an *EML4-ALK* fusion gene in NSCLC has been reported for the first time in 2007 [3]. In addition, *KIF5B*, *KLC1* and *TFG* have also been described as fusion partners [4]. Injection of *EML4-ALK* overexpressing 3T3 cells into nude mice induced tumor growth indicating transforming activity of the EML-ALK fusion protein [3]. *ALK* rearrangements have been detected in approximately 4–7% of the NSCLC patients [3,5]. The frequency is higher in young, non-smoking patients with adenocarcinoma [6]. The *EML4-ALK* fusion results in overexpression of the fusion product that includes the tyrosine kinase activity domain of *ALK* [7].

Despite an initial favorable response to crizotinib, patients inevitably acquire resistance due to selective pressure of the tyrosine kinase inhibitor (TKI) [1]. Different genomic aberrations have been identified as resistance mechanisms to ALK-TKI, including ALK-dependent and ALK-independent mechanisms. ALK-dependent mechanisms include gatekeeper (L1196M) or other mutations such as C1156Y and G1269A in the *ALK* kinase domain and *ALK* copy number gain [8–9]. Gatekeeper mutations are defined as mutations in the gatekeeper residue of the tyrosine kinase protein, i.e. the leucine residue at position 1196 [8]. ALK-independent mechanisms include *KRAS* and *EGFR* mutations (L858R) and *KIT* amplification. In addition, *AXL* overexpression and changes in the pathways of the epithelial-mesenchymal transition (EMT) have been described as a resistance mechanisms to the ALK-TKI in cell lines [10]. Despite the increasing number of known resistance mechanisms, the mechanisms remains unknown in approximately 18–44% of the patients [1,9].

As it is known that TKs can be activated by chromosomal translocations, we speculate that fusion genes might form a potential novel resistance mechanism. In this study we aimed to identify presence of fusion genes as a novel resistance mechanism in patients progressing on crizotinib using transcriptome sequencing. We used deFuse and EricScript to detect fusion genes in paired-end RNA sequencing (RNA-seq) data and validated fusion genes by RT-PCR and Sanger sequencing. Fusion genes confirmed in post-treatment samples were subsequently analyzed in the pre-treatment samples. In addition, we used the RNA sequencing data to determine presence of crizotinib resistance-associated mutations in *EGFR*, *KRAS* and *ALK* genes.

## Materials and Methods

### Patients and tumor samples

Patients were selected at our outpatient clinic of the University Medical Center Groningen when they had non-squamous cell lung cancer with an *ALK* break as determined by FISH (> 15% breaks). Among 36 *ALK*-positive NSCLC patients treated between 2010 and 2013, we had frozen tissue available of crizotinib-resistant post-treatment tumor samples of three lung adenocarcinoma patients (Table 1). Formalin fixed paraffin embedded (FFPE) tumor tissue was available before and after crizotinib treatment for all three patients. A normal lung tissue sample was used as control for the RT-PCR.

**Table 1:** Patients’ characteristics and fusion products detected in crizotinib resistant tumors.

Patient	Biobank no.	Sample accession ID at ENA website	Age at diagnosis	Smoking	Tumor response (PFS in months)	Type	Tumor (%)	High confidence gene fusions	Predicted ORF
#1	1211987	SAMEA3881068	27	None	PR (7.0)	Frozen	90	*EML4-ALK*	**Yes**
								*NRG1-RBPMS*	No
#2	1219581	SAMEA3881069	55	Current	PR (9.5)	Frozen	70	*EML4-ALK*	**Yes**
#3	1305996	SAMEA3881070	34	None	PR (15.9)	Frozen	90	*EML4-ALK*	**Yes**
								*CLIP4-VSNL1*	**Yes**
								*MCFD2-CLIP4*	No
								*KIAA0040-RFWD2*	No

PFS is progression free survival; ORF: Open reading frame.

### Informed Consent and Ethics

Written informed consent for tumor tissue from all three patients was obtained before biobanking and retrieval from the Groningen Pathology biobank. All patient data were anonymized and de-identified prior to analysis (Table 1). The authors were not informed about identification variables. The study was approved by the Medical Ethical Committee of the University Medical Center Groningen and conducted in accordance with the provisions of the Declaration of Helsinki and Good Clinical Practice guidelines. Due to the retrospective nature of this study, under Dutch Law for human medical research (WMO), no specific permission was compulsory from the Institutional Review Board.

### Fluorescence in situ hybridization (FISH)

FISH was performed using the *ALK* dual color break probes (Vysis LSI ALK Break Apart FISH Probe Kit, Abbott Molecular Inc., Des Plaines, USA) and *EML4-ALK* fusion FISH (Kreatech, Leica Biosystems, Wetzlar, Germany) following standard protocols. After deparaffinization, slides were incubated in TRIS/EDTA pH9.0 buffer in a pressure cooker for 7 min at 120°C. This was followed by an RNase (Thermo Fisher Scientific Inc., Waltham, USA) treatment step for 10 min at 37°C, followed by a pepsin (Sigma-Aldrich, St. Louis, United States) treatment for 1h at 37°C. Hybridization and wash steps were performed according the manufacturer´s protocol. Slides were mounted in vectashield with DAPI (1:1 diluted in vectashield). Three images were captured from each slide using an appropriate single filter (Olympus DP50 camera, USA). Scoring was performed according to the international guidelines (www.Abbott.com) by two independent well-trained and experienced readers and a case was called *ALK*-break positive if ≥15% of the evaluated neoplastic nuclei (n = 100) had a break-apart pattern. For the *EML4-ALK* fusion a case was called positive when >15% of the cells showed co-localization of the two FISH signals.

### ALK immunohistochemistry

ALK immunohistochemistry (IHC) was performed on 3 micron FFPE tumor tissue sections, using the ALK rabbit monoclonal antibody clone D5F3 (Roche, Basel, Switzerland) in the VENTANA BenchMark ULTRA according to the manufacturer’s protocol (Ventana, Tucson, Arizona). The staining was visualized using the OptiView DAB IHC Detection Kit (Ventana) and OptiView Amplification Kit (Ventana). Samples were scored ALK-positive if strong granular cytoplasmic brown staining present in the neoplastic cells [11]. Appropriate positive and negative controls were included in each experiment.

### RNA and DNA isolation

Total RNA was isolated from frozen tissue according to a standard laboratory protocol using TRIzol (Life technologies, Carlsbad, USA). RNA from FFPE samples was isolated using the RNeasy FFPE kit according to the manufacturer’s protocol (Qiagen, Venlo, The Netherlands). Genomic DNA from frozen tissue samples was isolated using a routine salt-chloroform protocol using standard protocols. The ReliaPrep^™^ FFPE gDNAMiniprep System kit (Promega, Madison, USA) was used to isolate DNA from FFPE samples following the protocol of the manufacturer. The NanoDrop (Thermo Fisher Scientific Inc., Waltham, USA) was used to determine DNA and RNA concentrations.

### Transcriptome sequencing and fusion detection

Library preparation for paired-end RNA sequencing was performed using the TruSeq RNA kit (Illumina, San Diego, USA), starting from 500ng of total RNA. Paired-end reads of 100nt were generated on the Hiseq2500 (Illumina, San Diego, USA). We used two independent algorithms to predict presence of the fusion transcripts. DeFuse (v.0.6.1) [12] and EricScript [13]. DeFuse maps the reads to the reference genome using an automated process which involves SAMtools [14], bowtie [15], BLAT [16] and GMAP [17]. EricScript uses a series of alignment steps, by BWA [18] and BLAT, to identify and precisely map discordant reads that point to gene fusions, after which the RNA-seq data are screened for the presence of spanning reads to support these putative fusions. We excluded fusions derived either from read-through transcripts or fusion genes that mapped to multiple genomic loci with high homology. We next focused on the fusion genes detected by both deFuse and EricScript. We inspected mapping of split reads and spanning reads using the University of California Santa Cruz (UCSC) genome browser. Predictions of the presence of an ORF in the fusion products were obtained from deFuse. RNA-seq data have been deposited on European Nucleotide Archive (ENA) website and are available under accession number: PRJEB12854.

### Validation of the fusion products by RT-PCR

cDNA was synthesized with Superscript II reverse transcriptase and random primers according to the company instruction starting from 500ng total RNA (Invitrogen, Carlsbad, USA). PCR was performed using 10ng cDNA as input in a final volume of 30μl containing 1x PCR buffer and MgCl_2_ (final concentration 1.5mM), 0.2μl Tag DNA polymerase (5unit/μl) (Invitrogen, Carlsbad, USA) and 500nM primers designed using Clone Manager Suite (Sci-Ed Software, Morrisville, USA) (Table in S1 Table). Amplification consisted of 35 (frozen samples) or 45 (FFPE) cycles using a thermocycler (Bio-Rad, Hercules, USA). PCR products were analyzed on a 3% agarose gel, purified using Zymoclean^™^ Gel DNA Recovery Kit (Zymo research, Irvine, USA) and sequenced at LGC Genomics (Berlin, Germany). Agarose gel pictures were captured using Gel Doc XR+ System (Bio-Rad, Hercules, USA).

### Identification and validation of mutations in *ALK*, *EGFR* and *KRAS* gene

For each patient the RNA-seq bam file, generated by RSEM (1.2.9) was inspected in IGV [19]. All exons of *ALK*, *EGFR* and *KRAS* genes were visually screened for coverage and the presence of known resistance-associated mutations. To validate *ALK* mutations, 50ng of DNA was amplified as described above using primers designed with Clone Manager Suite (Sci-Ed Software, Morrisville, USA) (S1 Table). M13F or M13R tails were added to the 5’ end of the primers designed for DNA to allow direct sequencing of the PCR products. Purification and sequencing was performed as described above. One of the *ALK* mutations was validated at the RNA level by RT-PCR and Sanger sequencing using a primer set that allowed specific amplification of the *EML4*-*ALK* breakpoint region.

### Detection of *ALK* C1156Y and G1269A mutations by droplet digital PCR (ddPCR)

Mutant and wild type ddPCR primers and probes to detect C1156Y and G1269A *ALK* gene mutations were obtained from Bio-Rad (Hercules, USA). The ddPCR was performed on 18ng of genomic DNA as measured by Qubit (Life technologies, Carlsbad, USA) according to the manufacturer’s instruction (Bio-Rad, Hercules, USA). Briefly, 11μl ddPCR Supermix for probes, 1μL of the mutation assay and genomic DNA were mixed in a final volume of 20μl. Droplets were generated using the QX100 Droplet generator after addition of 70μl droplet generation oil (Bio-Rad, Hercules, USA). PCR was performed on a T100 Thermal Cycler (Bio-Rad, Hercules, USA) using the following cycling conditions: 10 minutes at 95°C, 40 cycles of 95°C for 30 seconds, 55°C for 1 minute followed by 98°C for 10 minutes (ramp rate 2.5°C/sec). Samples were transferred to the QX200 Droplet Reader (Bio-Rad, Hercules, USA) for fluorescent measurement of FAM and HEX probes and data were analyzed by Quantasoft software version 1.6.6 (Quantasoft, Prague, Czech Republic). In addition to the pre- and post-treatment tumor samples, 10 normal control samples were used as negative controls. Sensitivity of the assays was 0.1 and 0.5% for C1156Y and G1269A respectively, as determined on dilution series of the post-treatment tumor samples in combination with the total number of droplets that could be analyzed in the primary tumor sample.

## Results

### Patients

The three patients, aged 27 to 56 years, were all tested positive for *ALK* IHC before crizotinib treatment (pre-treatment) and at disease progression (post-treatment). All three patients were *ALK* FISH positive both before crizotinib and at disease progression. Only patient #3 showed extra *ALK* copies in the diagnostic FISH analysis. In addition, *EML4-ALK* specific FISH revealed only one copy of this fusion per cell in this patient. Patient #1, #2 and #3 showed a partial response (PR) with progression free survival (PFS) of 7.0, 9.5 and 15.9 months, respectively (Table 1). Patient #1 was diagnosed with adenocarcinoma in March 2011 and treated with two courses of cisplatin and pemetrexed in the same month. She received crizotinib from October 2011 and died in December 2012. Patient #2 was diagnosed with lung adenocarcinoma in March 2010 and received cisplatin and pemetrexed in December 2011. He received crizotinib from January 2012 and one year later switched to ceritinib. Treatment was ended in October 2013 and the patient died in January 2014. Patient #3 was diagnosed with adenocarcinoma in May 2005 and received cisplatin and pemetrexed until June 2006, when a bilateral adnexectomy was performed for a large metastasis. In May 2008 she developed liver metastases and was treated with a single agent pemetrexed. She received crizotinib from November 2011 based on an *ALK*-positive FISH in the primary tumor sample and had a near complete response. In January 2013 she developed liver metastasis, which were treated with metastasectomy and radiofrequency ablation. In July 2013, she started treatment with ceritinib and had a complete response. Since then she is well and alive on maintenance ceritinib.

### Detection of fusion products

A total of 19.9, 19.9 and 28.9 million reads were aligned for post-treatment tumor samples of patient #1, #2 and #3, respectively. Seven fusion gene products were identified in these three tumor samples, including an *ALK* fusion gene in each patient (Table 1). The fusion partner was *EML4* in all three patients according to the deFuse and EricScript analysis. The breakpoint was in intron 20 of the *EML4* gene in patient #1 and intron 6 of the *EML4* gene in patients #2 and #3. The *EML4* gene was fused to exon 20 of the *ALK* gene in all three patients.

**Fig 1:**
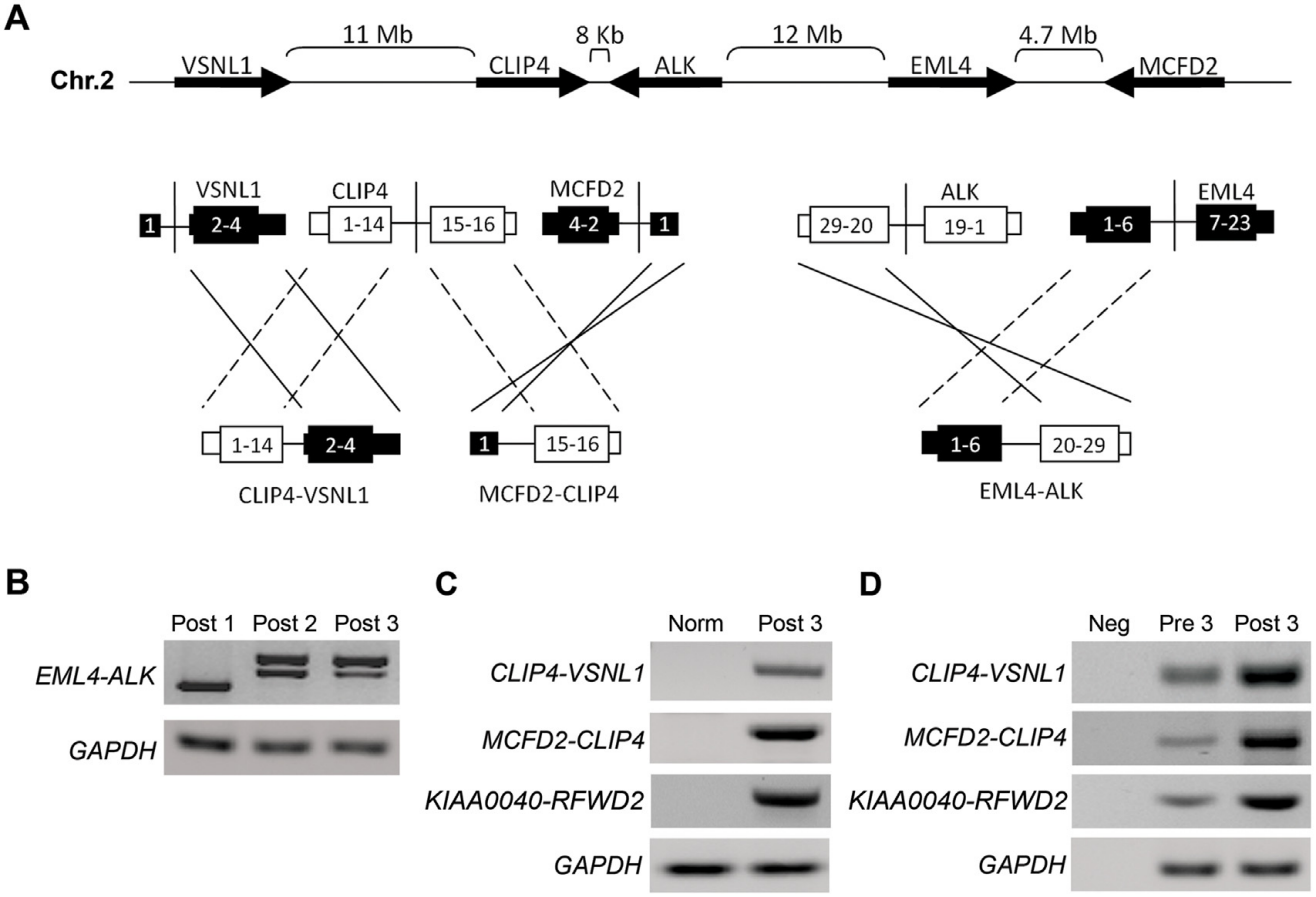
Schematic representation of fusion gene products clustered at the *ALK* locus and selected fusions validation. **(A)** Three fusion products clustered at a 25Mb genomic region including the *ALK* gene locus in the tumor of patient #3. Two of the three fusion products are the result of an inversion (*EML4-ALK* and *MCFD2-CLIP4*), whereas the third fusion product is generated via an eversion (*CLIP4-VSNL1*). *EML4-ALK* and *CLIP4-VSNL1* contain a predicted ORF. **(B)** Detection of *EML4-ALK* fusion in three crizotinib post-treatment tumor samples (post 1, post 2 and post 3, corresponding to post-treatment samples of patient #1, #2 and #3 respectively). **(C)** Validation of three novel fusion genes in frozen post-treatment tumor sample of patient #3. **(D)** Detection of the fusion genes in FFPE samples of post-treatment samples and analysis of the fusion gene in pre-treatment tumor sample of patient #3. Norm: Normal lung tissue; Pre: pre-treatment tumor sample; Post: post-treatment tumor sample; Neg: Negative control.

In patient #1, one additional fusion gene, i.e. *NRG1-RBPMS*, without a predicted ORF according to deFuse was detected. In patient #2, no additional fusion products were identified. Patient #3 contained three additional fusion genes, one with and two without predicted ORFs (Table 1). Two of the fusion genes (*CLIP4-VSNL1* and *MCFD2-CLIP4*) were the result of multiple genomic aberrations at the *ALK* gene region on chromosome 2 (Fig 1A). Both fusion products involved the *CLIP4* gene mapping 8kb downstream of the *ALK* gene. In one fusion transcript, exon 14 of the *CLIP4* gene was fused to exon 2 of the *VSNL1* gene, resulting in a fusion transcript with a predicted ORF. In the second fusion transcript, exon 15 of the *CLIP4* gene was fused to the non-coding exon 1 of the *MCFD2* gene. This fusion did not have a predicted ORF.

### Validation of the fusion products by RT-PCR

*EML4*-*ALK* fusion transcripts were confirmed by RT-PCR followed by Sanger sequencing in post-treatment tumor samples of patients #1, #2 and #3 (Fig 1B). We failed to validate the *NRG1-RBPMS* fusion in patient #1 on both the frozen and FFPE post-treatment sample, despite good amplification product for the house keeping gene (data not shown). This might be due to low expression level, or to design of a suboptimal primer sets, precluding efficient amplification. We next validated the three novel fusion products identified in patient #3. A PCR product of the expected size was observed for all three fusion genes in the frozen biopsy of the post-treatment tumor samples (Fig 1C). Sanger sequencing of these RT-PCR products confirmed the expected sequence consistent with the prediction of deFuse and EricScript. Next, we evaluated whether these fusion transcripts were also present in the pre-treatment FFPE tumor samples of these patients. FFPE samples of the post-treatment tumors were included as positive controls. The *CLIP4-VSNL1*, *MCFD2-CLIP4* and *KIAA0040-RFWD2* fusion transcripts were detected in the pre- and post-treatment tumor sample of patient #3 (Fig 1D).

### Identification and validation of mutations in *ALK*, *EGFR* and *KRAS*

**Fig 2:**
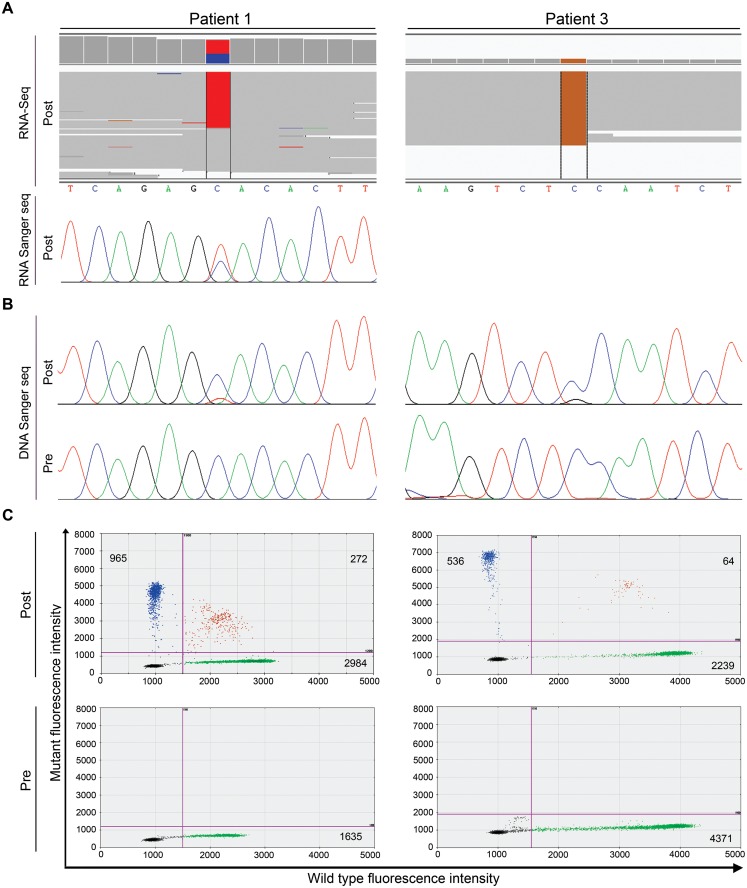
Detection of *ALK* gene mutations in tumor samples. **(A)** RNA-sequencing reads of the two mutations. Grey bars show the wild type positions, the colored bar indicates the mutant position. The number of wild type and mutant reads were 56/75 for patient #1 (c.3467G>A) and 0/25 for patient #3 (c.3806G>C)(Top). RNA Sanger sequencing in the post-treatment tumor sample of patient #1 confirmed presence of wild type and mutated *EML4-ALK* copy using primers covering the *ALK* break (Bottom). The sequences in this picture are based on plus strand, whereas the *ALK* gene is located on the minus strand of chromosome 2. **(B)** DNA Sanger sequencing results in the pre- and post-treatment tumor samples. **(C)** ddPCR results of the pre-and post-treatment tumor samples of patient #1 and #3. Number of positive droplets for mutant and wild type alleles is written in each gate of the scatter plots. Sensitivity of the assays was 0.1 and 0.5% for C1156Y and G1269A, respectively. Fractional abundance for the mutant allele was 26% and 19.8% in the post-treatment tumors of patients #1 and #3, respectively. Pre: pre-treatment tumor sample; Post: post-treatment tumor sample.

Mutations in *ALK*, *EGFR* and *KRAS* have been reported to confer resistance against crizotinib. To determine presence of mutations in these genes in the three post-treatment samples, we inspected the RNA-seq bam files in IGV. In patient #1 no *EGFR* gene mutations were observed, whereas for *KRAS* the coverage was too low. Analysis of the paired-end RNA-seq data revealed no mutations in the *EGFR* and *KRAS* genes in the post-treatment samples of patients #2 and #3 (Table 2). A mutation was found in 57% of the RNA-seq reads in the *ALK* gene, i.e. p.C1156Y (NM_004304.3:c.3467G>A), in patient #1. Sanger sequencing of the RT-PCR product using *EML4*-*ALK* fusion gene specific primers confirmed presence of both wild type and mutant *EML4-ALK* fusion gene transcripts consistent with the RNA-seq data (Fig 2A). No mutations were observed in the *ALK* gene in the post-treatment sample of patient #2. In patient #3, an *ALK* mutation was observed in 100% of the RNA-seq reads, i.e. p.G1269A (NM_004304.3:c.3806G>C). Sanger sequencing confirmed presence of the mutations at the DNA level in the post-treatment tumors of both patients (Fig 2B). No mutations were observed in the *KRAS* and *EGFR* genes (Table 2).

**Table 2:** Summary of the diagnostic FISH, immunohistochemistry and the transcriptome analysis results.

Patient	Pre-treatment		Post-treatment					
	*ALK* FISH (%)	*ALK* IHC	*ALK* FISH (%)	*ALK* IHC	*ALK* Mutation	*EML4-ALK* duplication	*EGFR* mutation	*KRAS* mutation
#1	>15	+	>50	+	p.C1156Y	+*	WT	Unknown
#2	>50	+	>50	+	WT	none	WT	WT
#3	>15	+	>50	+	p.G1269A	none	WT	WT

WT: Wild type; ALK-IHC is either positive or negative using D5F3 antibody for immunohistochemistry in combination with the Optiview system.

*See discussion.

Analysis of the *ALK* mutations in the pre-treatment tumor samples of patients #1 and #3 by Sanger DNA sequencing revealed no mutations. To exclude presence of a minor clone with the *ALK* mutation in the pre-treatment tumor samples we performed ddPCR. In the pre-treatment samples no mutations were detected. In the post-treatment tumors, the fractional abundance of the corresponding mutant alleles was 26% and 19.8% in patients #1 and #3, respectively (Fig 2C).

## Discussion

*ALK*-break positive NSCLC patients respond to crizotinib in over 60% of cases, but after 9 to 12 months drug resistance develops in all patients [20]. In several studies mutations in the *ALK* gene or mutations in *KRAS* and *EGFR* in re-biopsies were observed as mechanisms of the resistance [1,9,21]. In this study we focused on detection of novel fusion products as possible resistance mechanisms to crizotinib using RNA-seq. In addition, we also evaluated the presence of hotspot mutations in *ALK*, *KRAS* and *EGFR* known to be associated with crizotinib resistance using the same RNA-seq data.

In patient #1, we confirmed the presence of the *EML4-ALK* fusion gene in the post-treatment sample taken from a tumor growing under crizotinib. One additional fusion gene without a predicted ORF was identified in this patient, but could not be confirmed by RT-PCR. An C1156Y *ALK* mutation was observed in approximately half of the RNA-seq reads in this patient. This suggests that the *ALK* mutation was gained after duplication of the *EML4*-*ALK* fusion or that the mutation is present only in a proportion of the tumor cells, while being wild type in the other tumor cells. The alternative explanation, i.e. gain of a de novo fusion gene in combination with gain of an *ALK* mutation on one of the two fusion genes seems unlikely. The mutation was not present in the pre-treatment biopsy using the sensitive ddPCR. In patient #3, we confirmed the presence of the *EML4-ALK* fusion gene in the post-treatment sample. Three additional fusion genes were detected, of which one had a predicted ORF. However, all three fusion genes were present in the pre-treatment sample, and thus not treatment induced. In addition, we observed an G1269A *ALK* mutation in the post-treatment tumor, which was not detectable in the pre-treatment tumor sample using ddPCR. Gain of an *ALK* mutation most likely caused the crizotinib resistance in patients #1 and #3. ALK-dependent crizotinib resistance mechanisms were thus involved in 2 of the 3 patients.

Functional analysis of the two observed resistance-associated mutations in Ba/F3 and NIH3T3 cells has proven their role in crizotinib resistance [9,21]. The G1269A mutation is located close to the crizotinib binding site and induces a stronger resistance towards crizotinib than the C1156Y mutation [9]. The relative quick appearance of crizotinib resistance in patient #1 may be due to the combination of different post-treatment mechanisms, the milder C1156Y mutation and the potential *EML4-ALK* duplication. Moreover, based on the normalized RNA-seq reads, this patient had a 2 to 3 fold higher expression level of the *ALK* fusion gene as compared to the two other patients. Thus, despite gain of the less effective mutation, *EML4-ALK* duplication and the higher expression level might also have contributed to the short PFS.

A number of studies have investigated mechanisms of resistance to crizotinib in post-treatment tumor samples of NSCLC patients. *ALK* mutations were the most commonly observed aberrations identified in post-treatment biopsies of 16 out of 51 (31%) patients [1,9,21–24]. We detected *ALK* mutations in 2 of the 3 patients. Using ddPCR we showed that these mutations were not detectable in pre-treatment biopsies that is in agreement with the fact that these mutations are associated with resistance to crizotinib. *ALK* gain has been reported as resistance mechanism in 4 out of 36 (11%) patients [1,9,23]. We observed *EML4*-*ALK* RNA-seq reads with and without the *ALK* mutation in patient #1. This might indicate a mixed tumor cell population or duplication of the fusion gene with gain of an *ALK* mutation in one of the two copies of the *EML4*-*ALK* fusion gene. Of the 36 patients studied for both *ALK* mutations and *ALK* gain, only one case was positive for both.

In patient #2, we confirmed presence of the *EML4-ALK* fusion gene in the post-treatment sample. No additional fusion genes were identified. We did not find *ALK* mutations or gain of *ALK* copies, indicating the occurrence of an ALK-independent resistance mechanism. Also, we did not find evidence for the other currently known ALK-independent crizotinib resistance-associated aberrations in this patient. As the number of aligned reads in this patient was similar to patient #1 and we did detect the *EML4*-*ALK* fusion gene, it seems unlikely that we failed to detect other fusion genes. Moreover, we found no evidence of increased expression of *ALK* or *EGFR* in the RNA-seq data (results not shown). Other currently unknown ALK-independent resistance mechanisms might have been induced in this tumor sample.

In patient #3, three novel fusion gene products (one with and two without a predicted ORF) were present in both the pre- and post-treatment tumor samples. Given the gain of a functionally confirmed *ALK* mutation, it seems less likely that these fusions are associated with resistance to crizotinib. Moreover, these fusion products were already present in the pre-treatment tumor sample. The role of the three novel fusion gene products, one with and two without a predicted ORF in patient #3, remain unknown. The clustering of three fusion gene products within the *ALK* gene region suggests that this genomic region is an unstable region in advanced NSCLC. The frequent loss of (parts of) the short arm of chromosome 2 (2p14-16, 2p23.3 and 2p24.3) as observed in NSCLC is consistent with this region being susceptible to chromosomal breaks [25–26]. Based on the orientation of the genes, the FISH results and the two breakpoints in the *CLIP4* gene, it is most likely that the *CLIP4-VSNL1* and *MCFD2-CLIP4* are present on the same chromosome as a result of a duplication followed by an inversion. The *ELM4-ALK* fusion gene might be present on the same or on the sister chromosome.

The question is what can be done for patients that become resistant to crizotinib. Besides, crizotinib and ceritinib that both show high tumor response rates, next generation ALK inhibitors such as alectinib, brigatinib (AP26113) and lorlatinib (PF-06463922) are under development and show high response rates in diverse resistance associated ALK mutants. For instance, ceritinib is active against crizotinib resistant ALK mutant forms such a L1196M, G1123S, G1269A, S1206Y and I1171T. Alectinib is active against L1196M, C1156Y, 1151T-ins, L1152R, F1174L, G1269A, and R1275Q. Brigatinib is active against L1196M, F1174L, G1269A, but not S1206Y. PF-6463922 is active against all the above-mentioned ALK mutant forms [27–30]. A recent study on a single patient with NSCLC has shown that crizotinib-resistant *ALK*-positive cells can be resensitized to crizotinib after treatment with loratinib via acquiring *ALK* L1198F mutation [31].

In conclusion, we identified four novel gene fusion products in two of the three crizotinib resistant post-treatment tumor samples. In two patients gain of *ALK* mutations was the most likely resistance mechanisms. In the third patient, the putative ALK-independent resistance mechanism remained unclear. Overall, it is unlikely that the fusion genes identified in our study are involved in resistance to crizotinib.

## Supporting Information

S1 Table List of primers for detection of fusion transcripts and *ALK* mutations in frozen and FFPE samples. (DOCX)
