# Supplementary material for: Genomic Aberrations in Crizotinib Resistant Lung Adenocarcinoma Samples Identified by Transcriptome Sequencing
Source: PLoS One. 2016 Apr 5;11(4):e0153065. doi: 10.1371/journal.pone.0153065 (PMC4821611; doi:10.1371/journal.pone.0153065)
Supplement: S1 Table — (DOCX) [file pone.0153065.s001.docx]

**S1 Table.** List of primers for detection of fusion transcripts and *ALK* mutations in frozen and FFPE samples.

| **Primer set** | **Name** | **Primer sequence** | **DNA/RNA** | **Used in** | **Annealing (°C)** | **No. of cycles** | **Product size (bp)** | **Transcript Ensembl ID** |
| --- | --- | --- | --- | --- | --- | --- | --- | --- |
| 1 | EML4-E5-F | 5’-ACGACCATCACCAGCTGAAA-3’ | RNA | Frozen | 55 | 35 | 327/294 | ENST00000318522 |
|  | ALK-E20-R | 5’-CTGATGGAGGAGGTCTTGCC-3’ |  |  |  |  |  | ENST00000389048 |
| 2 | EML4-E20-F | 5’-CAGATATGGAAGGTGCACTG-3’ | RNA | Frozen | 55 | 35 | 252 | ENST00000318522 |
|  | ALK-E20-R | 5’-CTGATGGAGGAGGTCTTGCC-3’ |  |  |  |  |  | ENST00000389048 |
| 3 | EML4-E20-F | 5’-GTCTAACTCGGGAGACTATG-3’ | RNA | Frozen | 60 | 35 | 413 | ENST00000318522 |
|  | ALK-E23-R | 5’-CCCCAATGCAGCGAACAATG-3’ |  |  |  |  |  | ENST00000389048 |
| 4 | ALK-E22-F | 5’-GTAAAACGACGGCCAGTTGGCTTGCGGACTCTGTAG-3’ | DNA | Frozen/FFPE | 60 | 35 | 170 | ENST00000389048 |
|  | ALK-E22-R | 5’-GGAAACAGCTATGACCATGGGTGAGGGTGTCTCTCTGTG-3’ |  |  |  |  |  | ENST00000389048 |
| 5 | ALK-E25-F | 5’-GTAAAACGACGGCCAGCTTCCCAGAGACATTGCTGC-3’ | DNA | Frozen/FFPE | 58 | 35 | 163 | ENST00000389048 |
|  | ALK-E25-R | 5’-GGAAACAGCTATGACCATGCCATTCTTGAGGGGCTGAGG-3’ |  |  |  |  |  | ENST00000389048 |
| 6 | CLIP4-E13-F | 5’-ATGGTTCAGTTGGAGGTGTG-3’ | RNA | Frozen | 55 | 35 | 338 | ENST00000320081 |
|  | VSNL1-E3-R | 5’-CTTGGAGGCGTCTCCATAAG-3’ |  |  |  |  |  | ENST00000404666 |
| 7 | MCFD2 -E1-F | 5’-AGCCGAGGAAGAGCGTTTTG-3’ | RNA | Frozen | 55 | 35 | 380 | ENST00000319466 |
|  | CLIP4-E16-R1 | 5’-ATAGCGCTTGTCACCCACTG-3’ |  |  |  |  |  | ENST00000320081 |
| 8 | KIAA0040-E3-F1 | 5’-GAACGTGACCTCCAGGAAAG-3’ | RNA | Frozen | 55 | 40 | 356 | ENST00000444639 |
|  | RFWD2-E13-R | 5’-TGACCTCTGTCCTGTGAATC-3’ |  |  |  |  |  | ENST00000367669 |
| 9 | CLIP4-E14-F | 5’-GATTCCCTGGATACCCTTTC-3’ | RNA | FFPE | 55 | 40 | 88 | ENST00000320081 |
|  | VSNL1-E2-R | 5’-CCAGTTTGCTATTCTGCTTC-3’ |  |  |  |  |  | ENST00000404666 |
| 10 | MCFD2 -E1-F | 5’-AGCCGAGGAAGAGCGTTTTG-3’ | RNA | FFPE | 55 | 45 | 123 | ENST00000319466 |
|  | CLIP4-E15-R2 | 5’-GGGAAGAAGCAGAAGTTGTG-3’ |  |  |  |  |  | ENST00000320081 |
| 11 | KIAA0040-E3-F2 | 5’-TGACAACGCAAAGCAAGAAG-3’ | RNA | FFPE | 55 | 45 | 116 | ENST00000444639 |
|  | RFWD2-E12-R | 5’-CAGCAATCGCAAAATAGTCAC-3’ |  |  |  |  |  | ENST00000367669 |

Underlined nucleotides: M13 primers
